# CD4^+^-mediated colitis in mice is independent of the GPR183 and GPR18 pathways

**DOI:** 10.3389/fimmu.2022.1034648

**Published:** 2022-10-28

**Authors:** Martina Dicker, Yingcong Li, Daniel A. Giles, Greet Verstichel, Viankail Cedillo Castelan, Gabriel Ascui-Gac, Ting-Fang Chou, Tamara Perez-Jeldres, Hilde Cheroutre, Mitchell Kronenberg

**Affiliations:** ^1^ Center for Autoimmunity and Inflammation, La Jolla Institute for Immunology, La Jolla, CA, United States; ^2^ Department of Molecular Biology, University of California, San Diego, La Jolla, CA, United States

**Keywords:** inflammatory bowel disease, chemokine receptor, gene expression, T cell transfer model, DSS model

## Abstract

Colitis is characterized by an exacerbated intestinal immune response, but the genetic and other mechanisms regulating immune activation remain incompletely understood. In order to identify new pathways leading to colitis, we sought to identify genes with increased expression in the colons of patients that also are near loci identified by genome wide association studies (GWAS) associated with IBD risk. One such SNP, rs9557195 was of particular interest because it is within an intron of *G-protein-coupled receptor (GPR) 183*, known to be important for lymphocyte migration. Furthermore, this SNP is in close proximity to the gene encoding another G-protein coupled receptor, *GPR18*. Analyzing publicly available datasets, we found transcripts of *GPR183* and *GPR18* to be increased in colon biopsies from ulcerative colitis and Crohn’s disease patients, and *GPR183* was even more increased in patients resistant to TNF treatment. Expression of both genes also was increased in mouse models of colitis. Therefore, our aim was to understand if increased expression of these GPRs in the intestine is related to disease severity in colitis models. Here we investigated the role of these receptors in the T cell transfer model and the dextran sulfate sodium model. In the T cell transfer model, GPR183 expression on donor T cells, as well as on other cell types in the *Rag^-/-^* recipients, was not essential for severe colitis induction. Furthermore, deficiency in *Rag^-/-^* mice for the enzyme that synthesizes a cholesterol metabolite that is a major ligand for GPR183 also did not affect disease. Similarly, lack of GPR18 expression in T cells or other cell types did not affect colitis pathogenesis in the T cell transfer or in the dextran sulfate sodium model. Therefore, despite increased expression of transcripts for these genes in the intestine during inflammation in humans and mice, they are not required for disease severity in mouse models of colitis induced by chemical injury or T cell cytokines, perhaps due to redundancy in mechanisms important for homing and survival of lymphocytes to the inflamed intestine.

## Introduction

The intestinal immune system has to be carefully balanced to tolerate dietary antigens and the commensal microbiome, while efficiently defending from invading pathogens. Therefore, imbalances of these regulatory mechanisms, resulting in part from genetic defects, manifest in inflammatory bowel diseases (IBD), such as Crohn’s disease and ulcerative colitis (UC). GWAS studies have mapped more than 200 loci related to IBD susceptibility. It has been difficult to associate polymorphisms with disease-causing mechanisms, as they could affect distal genes or only affect gene expression in a few cell types. In order to understand the mechanisms underlying IBD risk derived from GWAS studies, and to identify new targets associated with IBD, we sought to identify genes with increased expression in the diseased tissues of IBD patients that also are associated with loci identified from GWAS studies. Based on this strategy, we focused on the single nucleotide polymorphism (SNP) rs9557195 on human chromosome 13, linked to both Crohn’s disease and UC (1). This SNP is located within an intron of the *G-protein-coupled receptor 183 (GPR183)*. GPR183 was discovered in 1993 as an Epstein-Barr virus-induced orphan receptor in Burkitt lymphoma cell lines (1), hence its previous name is Epstein-Barr virus-induced G-protein coupled receptor 2 (EBI2). It was de-orphanized in 2011 (2, 3), in work showing that GPR183 ligands are dihydroxylated oxysterols resulting from cholesterol oxidation. The strongest affinity ligand is 7α,25-dihydroxycholesterol (7α-25-diHC), which is synthesized by sequential enzymatic reactions carried out by cholesterol 25-hydroxylase (CH25H) and cytochrome P450 family 7 subfamily member B1 (CYP7B1) (2, 3). GPR183 is of particular interest because it is expressed on many types of mouse and human lymphoid and myeloid cells (2–8). Furthermore, GPR183 is important for the localization of lymphocytes in various tissues (2, 3, 5–9), plays a role for inflammation in the mouse model of multiple sclerosis (9). While this study was underway, several groups focused on GPR183 in IBD, and its expression was found to be increased in inflammatory bowel disease patients (10). Although in dextran sodium sulfate (DSS) induced colitis *Gpr183* deficient mice were not different from the controls (10), other evidence suggests a role of GPR183 in colitis pathogenesis in mice stimulated by anti-CD40 to achieve innate immune activation (5)

The SNP rs9557195 also is in close proximity to the *GPR18* gene locus (11), which therefore is a candidate IBD susceptibility gene as well. From its discovery in the late 1990s GPR18 has been considered as an orphan receptor according to the International Union of Basic and Clinical Pharmacology (IUPHAR) (12). In humans and mice GPR18 expression was found to be abundant in spleen, thymus, lymph node, lung, peripheral blood and the intestine (13, 14). A higher expression of GPR18 was detected in mouse intestinal intraepithelial T cells compared to splenic T cells (15). Although GPR18 shares low sequence homology with the cannabinoid receptors CB1R and CB2R (~13% and 8%) it has been linked to the endocannabinoid system (ECS) due to interaction with a range of synthetic and endogenous cannabinoids (16–18). Another putative ligand of GPR18 is resolvin D2 (RvD2), an endogenous polyunsaturated fatty acid metabolite (13). GPR18 was shown to have distinct functions in several immune cells. GPR18 plays a role in BV2 microglial cell migration and proliferation (19, 20), neutrophil infiltration (13, 21, 22), and macrophage differentiation and efferocytosis (13, 22–24), and was previously found to regulate the population of γδ T cells in the small intestine (15).

Here we showed increased expression of the genes encoding both of these receptors and *CH25H*, important for the GPR183 ligand, in the intestine of IBD patients and mouse models of inflammation, with increased *GPR183* in patients resistant to TNF blockade. We also studied the roles of GPR18 and GPR183 in the T cell transfer model and GPR18 in the DSS model of colitis. We investigated the role of these G-protein-coupled receptors on T cells as well as on innate cells in the host.

We could demonstrate that neither receptor is essential for influencing disease severity in these models, despite increased expression during inflammation, suggesting that there are redundant mechanisms for maintaining inflammatory cell populations in the inflamed intestine.

## Materials and methods

### Animals

All mice were bred and housed under specific pathogen-free conditions at the La Jolla Institute for Immunology (La Jolla, CA). All mice were on the C57BL/6J background. *Gpr183^−/−^* and *Gpr18^−/−^* mice were kindly provided by Dr. Jason Cyster (UCSF, San Francisco, California), generated as described previously (15, 25). *Ch25h^−/−^* mice were purchased from the Jackson Laboratories (Bar Harbor, ME). *Rag1^−/−^* mice were bred in house and originated from Jackson Laboratories. *Rag1^−/−^Gpr183^−/−^, Rag1^−/−^Gpr18^−/−^ and Rag1^−/−^Ch25h^−/−^* mice were generated by crossing the indicated strains. Breeders of strains on the *Rag1^−/−^* background were housed using sterile caging and were treated for life with antibiotics, for one week on and one week off (Bactrim, 5 mL per 8 oz, Covetrus Vet Supplies) to make them less susceptible to infections by common bacteria in the vivarium. Experimental mice were not treated in this way. Mice were co-housed littermates and used for experiments at approximately 10-12 weeks of age. Mice of both sexes were analyzed initially, but male mice were used for all the experiments shown because of their greater body weight and resilience. All procedures were approved by the La Jolla Institute for Immunology Animal Care and Use Committee and are compliant with the ARRIVE standards.

### Experimental colitis models

#### Chronic DSS-induced colitis

Mice received 2.5% (w/v) DSS (molecular mass = 165.19g/mol,Thermofisher) in the drinking water for 2 cycles. As previously described, 1 cycle is comprised of 5 days of water plus DSS and 2 days with regular drinking water (26). Mice were littermates and animals in all experiments were of similar age (+/- 10 days). Body weight and appearance were monitored daily.

#### T cell transfer model

Typically, splenocytes from three to four donor mice were isolated, pooled and CD4 T cells were purified using EasySep Mouse CD4^+^ T cell isolation kit (STEMCELL Technologies). Naïve CD4 T cells were sorted for the CD4^+^CD45RB^high^ population using an BD FACSAria cell sorter. Donor strains included either C57BL/6, or *Gpr18^−/−^*, or *Gpr183^−/−^*. 2.5×10^5^ cells were injected into the bloodstream by the retro-orbital route into *Rag1^−/−^*, *Rag1^−/−^Gpr18^−/−^*, *Rag1^−/−^Gpr183^−/−^* or *Rag1^−/−^Ch25h^−/−^* recipients. A pool of 3 spleens provided enough cells for 10-12 recipients. Body weight and appearance were monitored 2-4 times per week. Mice were groups of littermates and animals in all experiments were of similar age (+/- 10 days in average).

For both colitis models, mice were euthanized *via* CO_2_ inhalation, in compliance with approved animal protocols, within 24 h of losing more than 20% of their starting body weight. Some mice died prior to euthanasia, all death are indicated in the graphs. For the transfer model, the experiments were ended by approximately six-eight weeks, or earlier if the number of mice that reached to or near the endpoint of 20% bodyweight loss affected the statistical number of mice in both groups

### Histology and scoring

#### DSS colitis

Upon endpoint the colon length was measured and a piece of distal colon and cecum were fixed in zinc formalin (Medical Chemical Corporation) for 24 hours. Samples were transferred to 70% isopropanol. Following paraffin embedding, fixed tissue was stained with hematoxylin & eosin (H & E). Images were generated from 4 sections per organ on an Axioscan-Z1 platform (Zeiss) using Zen-2.3 software. Slides were scored blindly according to the following criteria: inflammation, area of infiltration, crypt damage and edema, as described previously (27), except that four randomly selected areas were analyzed. A combined histological score obtained from adding the scores of the four sections was determined according to **Table 1**.

**Table 1:** Histology scoring of DSS model.

Inflammation	Area of infiltration	Crypt damage	Edema
0= none	0= none	0= none	0= none
1= mild	1= mucosa	1= basal 1/3 damaged	1= ≤2x submucosal thickness
2= moderate	2= mucosa & submucosa	2= basal 2/3 damaged	2= ≥2x submucosal thickness
3= severe	3= transmural	3= only surface epithelium intact	
		4= entire crypt and epithelium lost	

#### T cell transfer induced colitis

Upon termination of experiments the whole colon was cut in three parts from rectum to cecum. The fecal content was removed by flushing with PBS and the tissue was fixed with Zinc Formalin for 2 min. Colons were opened longitudinally, flattened, cut into 3 equal parts and further fixed in Zinc Formalin for 48h in cassettes. Samples were transferred to 70% isopropanol. Following paraffin embedding, fixed tissue was stained with H&E. Images were generated using an Axioscan-Z1 platform (Zeiss) and Zen-2.3 software. The middle section was utilized for scoring. Slides were scored blindly according to the following criteria: inflammatory infiltrate, submucosal infiltrate, crypt density, crypt hyperplasia, goblet cell loss and muscle thickening as described previously (28). Four randomly selected areas were analyzed and a combined histological score was determined as shown in **Table 2**.

**Table 2:** Histology scoring of T-cell transfer model.

Inflammatory infiltrate	Goblet cell loss	Crypt density	Crypt hyperplasia	Muscle thickening	Submucosal inflammation
0= none	0= none	0= normal	0= none	0= none	0= none
1= mild	1 = <10%	1= decreased by <10%	1= slight increase	1= slight	1= individual cells
2= moderate	2 = 10-50%	2= decreased by 10% - 50%	2 = 2-3 fold increase	2 = 2-3 fold increase	2= infiltrates
3= transmural	3= >50%	3= decreased by >50%	3= >3-fold increase	3= >3-fold increase	3= large infiltrates

### Colon explant cytokine quantification

Following T cell transfer, four 3 mm punch biopsies from distal colon were collected. Biopsies were weighed and cultured in 48-well plates with 500 μL RPMI containing 10% FBS for 6 hours. Supernatants were collected and analyzed using a cytometric bead array (CBA) and the mouse Th1/Th2/Th17 cytokine kit (BD Biosciences) and analyzed using a BD FACS Celesta analyzer and FCAP array software v3.0.

### Flow cytometry

Flow cytometry analysis was performed on a LSR Fortessa analyzer (BD Biosciences) and data was analyzed using FlowJo software (Tree Star).

### Bioinformatic analysis

A list of genes with probable expression alteration when in presence of risk associated loci for IBD was built using lists from two GWAS publications (1, 29) From (30), 38 loci were identified from a trans-ancestry meta-analysis. An additional 300 candidate genes from 125 loci detected, 39 of these candidate genes were supported by two or more methods (eQTL, GRAIL, DAPPLE or coding SNP annotation). The gene list was further filtered by an GWAS IBD-associated False Discovery Rate higher than 0.05. Publicly available datasets for mRNA from colon biopsies of UC and Crohn’s disease patients were retrieved from GSE16879. Microarray dataset for mouse colon tissue after T cell transfer model of colitis was retrieved from GSE27302. Microarray dataset for mouse colon tissue in response to DSS was retrieved from GSE22307.

Both mouse and human transcriptomic data were analyzed by DESeq2 package in R. A scRNA-seq dataset for CD4 and CD8 T cells PBMC from UC patients was retrieved from GSE125527 and analyzed in Seurat v4 in R.

### Statistical methods

Data are plotted as mean ± standard error of the mean (SEM), and statistical significance for weight loss graphs was determined by two-way ANOVA (Tukey’s test). Statistical significance for multiparameter comparisons with pooled experiments was determined by nonparametric T-test (Mann-Whitney test). Significance for human gene expression data was determined by ANOVA (Kruskal-Wallis test). One-way ANOVA (Tukey’s test) was used for mouse gene expression data and cytokine measurements.

### Data and software availability

All graphs and statistical analyses were generated using Prism 9 software (GraphPad Software, San Diego, CA).

## Results

### GPR183 expression in healthy and diseased individuals

Most GWAS hits are in non-coding regions of the genome, and therefore we analyzed for genes with increased mRNA expression in patient colon samples from both UC and Crohn’s disease patients whose expression related to a GWAS hit for IBD susceptibility by at least two methods. A relatively small list of genes associated with GWAS hits were significantly increased in colon of IBD patients (**Figures S1A, S1B**).

Among these, we found increased *GPR183* expression, not only in colon biopsies from UC but also from Crohn’s disease patients. Importantly, there was a further increase comparing patients with both forms of IBD that were resistant to TNF blockade (**Figures S1C, S1D**).

In a mouse model of colitis initiated by innate immune activation with anti-CD40 mAb, GPR183 was important for the localization of ILC and for inducing inflammation (5). In DSS induced colitis, however, which does not depend on T cells, GPR183 did not contribute to pathogenesis unless the mice were on the *Il10^-/-^* gene background (10). We found that *Gpr183* mRNA was slightly but significantly elevated in the colons of mice in the T cell transfer model of colitis (**Figure S1E**), which is highly dependent on adaptive immunity.

We analyzed the expression of *GPR183* in human lymphocytes using scRNA-seq data from PBMCs and distal colon biopsies of UC patients vs. healthy controls. *GPR183* was expressed in populations of B and T cells from colon and peripheral blood, with increased expression in B and T cell subsets from the colon of patients with disease (**Figures S2A, S2B**). Considering these results from mouse model and human patients, we determined if GPR183 might play a role in IBD pathogenesis through expression in T cells or in T cell-dependent models of inflammation.

### GPR183 expression does not affect T cell-mediated colitis

Because of the increased expression in colonic T cells from patients with IBD, we tested the effect of GPR183 deficiency in the T cell transfer model of colitis, in which CD4^+^ CD45RB^high^ (naïve) T cells, depleted of regulatory T cells, are adoptively transferred into *Rag1^-/-^* recipients.

In the hosts the donor T cells cause pancolitis and small bowel inflammation mediated by cytokines such as INFγ and IL-17A (30, 31).

**Figure 1:**
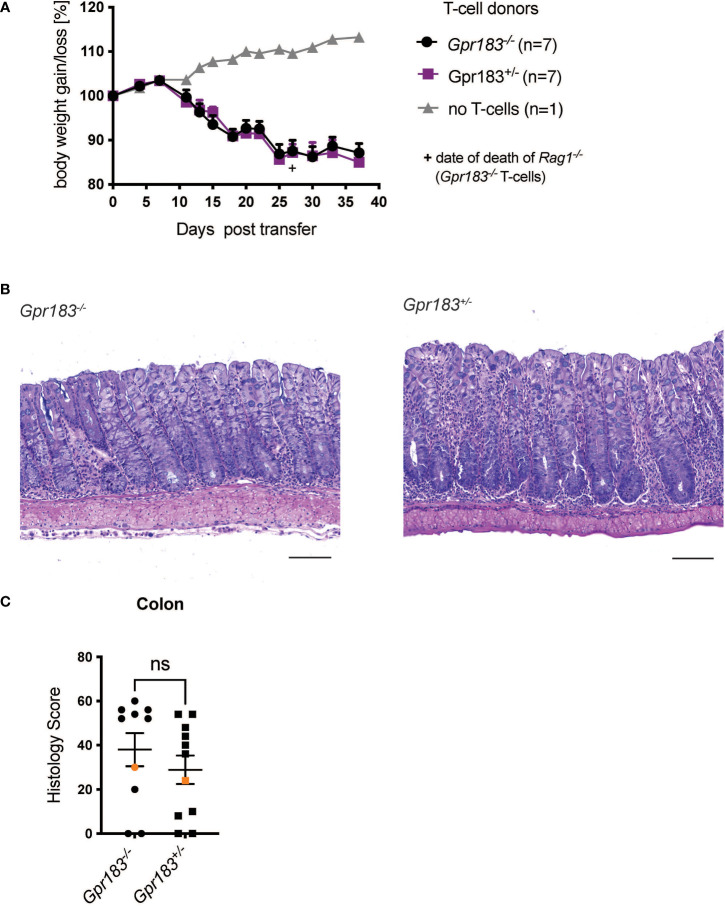
T cell expression of GPR183 does not play a role in the transfer colitis model. Colitis was induced by adoptive transfer of GPR183 deficient (*Gpr183^-/-^*) vs. littermate control (*Gpr183^+/-^*) naive CD4^+^ T cells into *Rag1^-/-^* recipients. **(A)** Weight change as % starting body weight was monitored on the indicated days. A representative weight graph from one of 2 independent experiments is shown. The weight of dead mice was carried forward, the date of death indicated with a + sign for one mouse for *Gpr183^-/-^* T cells. **(B)** H&E staining of representative colon sections (middle part of whole colon) at 40x (scale bar 100 μm) magnification **(C)** Blinded histology score from the middle part of whole colon of combined from the 2 independent experiments. Representative images in **(B)** were taken from mice represented by the orange colored data points. Error bars represent S.E.M. Two-way ANOVA (Tukey’s test) **(A)** and nonparametric T-test (Mann-Whitney test) **(C)**. ns, not significant.

We injected naïve CD4^+^ T cells from GPR183 deficient (*Gpr183^-/-^*) or control littermates (*Gpr183^+/-^*) into *Rag1^-/-^* recipient mice. We followed weight loss over approximately 5 weeks and did not find an effect of T cell GPR183 deficiency (**Figure 1A**). Furthermore, histopathologic scoring of the colon sections revealed that the colitis severity was similar when *Gpr183^-/-^* or control T cells were injected into *Rag1^-/-^* recipients (**Figures 1B, C**). Taken together, the expression of this G-protein coupled receptor by CD4^+^ T lymphocytes did not play an essential role in pathogenesis in the adoptive transfer model of colitis.

### GPR183 and CH25H expression by innate cells is not required for T cell transfer colitis

**Figure 2:**
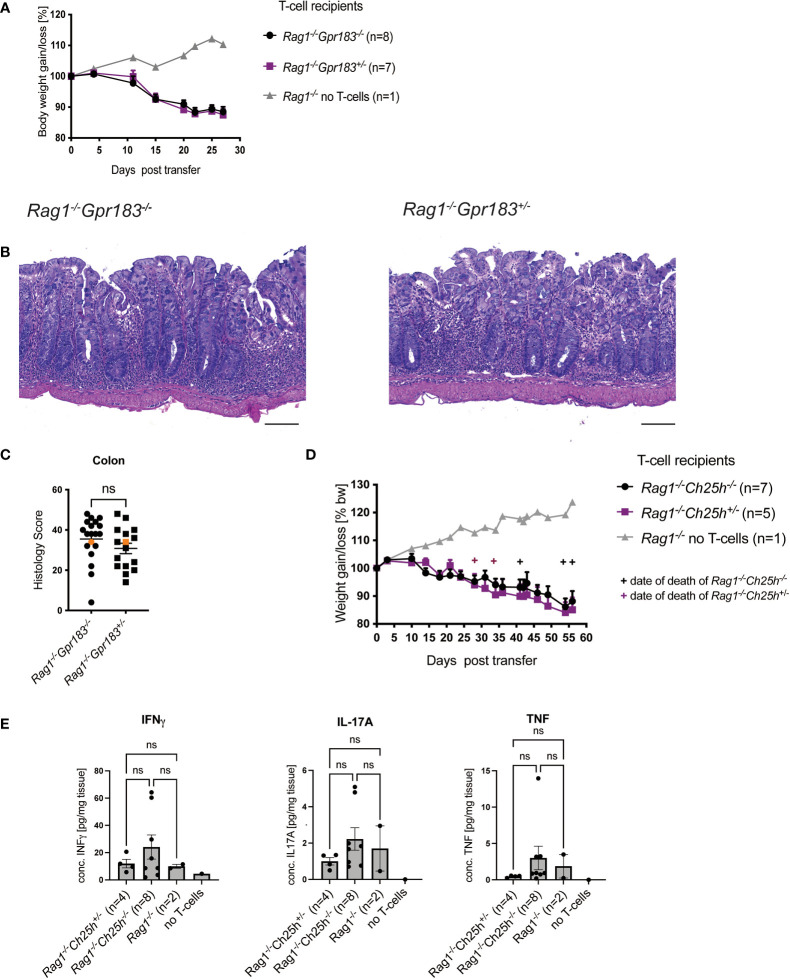
Expression of GPR183 or CH25H in Rag-/- mice are not required for colitis. Colitis was induced by adoptive transfer of naive CD4^+^ T cells from wildtype C57BL/6 mice into GPR183 deficient (*Gpr183^-/-^*) vs. littermate control (*Gpr183^+/-^*) *Rag1^-/-^* recipients. **(A)** Weight change was monitored as % starting body weight on the indicated days. A representative weight graph from one of 3 independent experiments is shown. **(B)** H&E staining of representative colon sections (middle part of whole colon) at 40x (scale bar 100 μm) magnification. **(C)** Blinded histology score from the middle part of whole colon combined from 3 independent experiments. Representative images in B) were taken from mice represented by the orange colored data points. **(D)** Colitis was induced by adoptive transfer of naive CD4^+^ T cells from wildtype C57BL/6 mice into CH25H deficient (*Ch25h^-/-^*) vs. control (*Ch25h^+/-^*) *Rag1^-/-^* recipients. Disease progression was monitored by assessing weight loss. A representative weight graph from one of 3 independent experiments is shown. The weight of dead mice was carried forward, indicated with + signs in magenta for control mice and in black for *Rag1^-/-^Ch25h^-/-^* mice. **(E)** Colon explant cytokine quantification from distal colon at the end of T-cell transfer model. Cytokines were measured by CBA; two independent experiments were pooled. Error bars represent S.E.M. Two-way ANOVA (Tukey’s test) (A and D) and nonparametric T-test (Mann-Whitney test) **(C)** and One-way ANOVA (Tukey’s test) **(E)**. ns, not significant.

Based on the literature, including the effects of GPR183 deficiency on Group 3 innate lymphoid cells (ILC3) in the anti-CD40 induced colitis model (5), we investigated the effect of GPR183 deficiency on innate immune cells and other cell types in the T cell transfer model. We crossed *Gpr183^-/-^* and control littermates onto a *Rag1^-/-^* background and injected wildtype naïve CD4^+^ T cells into GPR183-deficient (*Gpr183^-/-^*) versus littermate control (*Gpr183^+/-^*) *Rag1^-/-^* recipients. We did not observe an effect of absence of GPR183 expression on disease severity, measured by assessing weight loss of the *Rag1^-/-^Gpr183^-/-^* recipients and littermate control mice (**Figure 2A**). Additionally, histopathology scoring of the whole colon did not show a difference between *Rag1^-/-^* mice with the two genotypes (**Figures 2B, C**).

In parallel we determined if the production of the major GPR183 ligand, 7α,25-diHC, in *Rag1^-/-^* recipient was relevant for colitis pathogenesis. Therefore, we crossed *Ch25h^-/-^* mice onto a *Rag1^-/-^* background and tested the outcome following injection of wildtype naïve CD4^+^ T cells as described above. There was no difference in terms of disease progression as measured by weight loss in *Rag1^-/-^* recipients that did not express CH25H compared to those that did (**Figure 2D**). We also assessed cytokine production by colonic cells in organ fragment cultures from these mice. There were no differences in INFγ, IL-17A and TNF between *Rag1^-/-^ Ch25h^-/-^* and control mice (**Figure 2E**).

Overall, although expression of this G-protein coupled receptor was increased in the colon in mouse colitis models, our findings did not show an effect of GPR183 expression on T cell-mediated colitis in mice, neither when GPR183 expression was eliminated on T cells or when its expression by innate cells in the hosts was eliminated.

### GPR18 expression in healthy and diseased individuals

The gene encoding human *GPR18* is approximately 40 kb from the *GPR183* locus (**Figure S3A**) and SNP rs9557195 could affect GPR18 function or expression as well. Using publicly available microarray data of colon biopsies, we found that, like *GPR183*, *GPR18* transcripts also were increased in the colons of UC and Crohn’s disease patients (**Figure S3B**).

In contrast to *GPR183*, *GPR18* mRNA was not different comparing patients that did or did not respond to TNF blockade (**Figure S3C**). We analyzed published scRNA-seq data of UC patients vs. healthy controls to assess *GPR18* expression in human peripheral blood and matched colon biopsies. In PBMCs from patients and healthy controls, we could not detect expression of this receptor on naïve/memory, regulatory T cells and γδ T cells as well as B cell types (**Figure S3D**), although a low expression of *GPR18* may have missed detection. In cells derived from the distal colon biopsies, we detected increased *GPR18* mRNA in naïve B cells from patients compared to healthy controls, whereas it was not expressed by colonic T cell types from either group (**Figure S3E**).

Despite low expression of *GPR18* on lymphocytes, increased expression by other cell types could play a role in colitis pathogenesis, and therefore we tested a role for GPR18 in models of colitis dependent on innate immunity (DSS) and on adaptive immunity (T cell transfer model).

### GPR18 does not play a role in the DSS model of colitis

**Figure 3:**
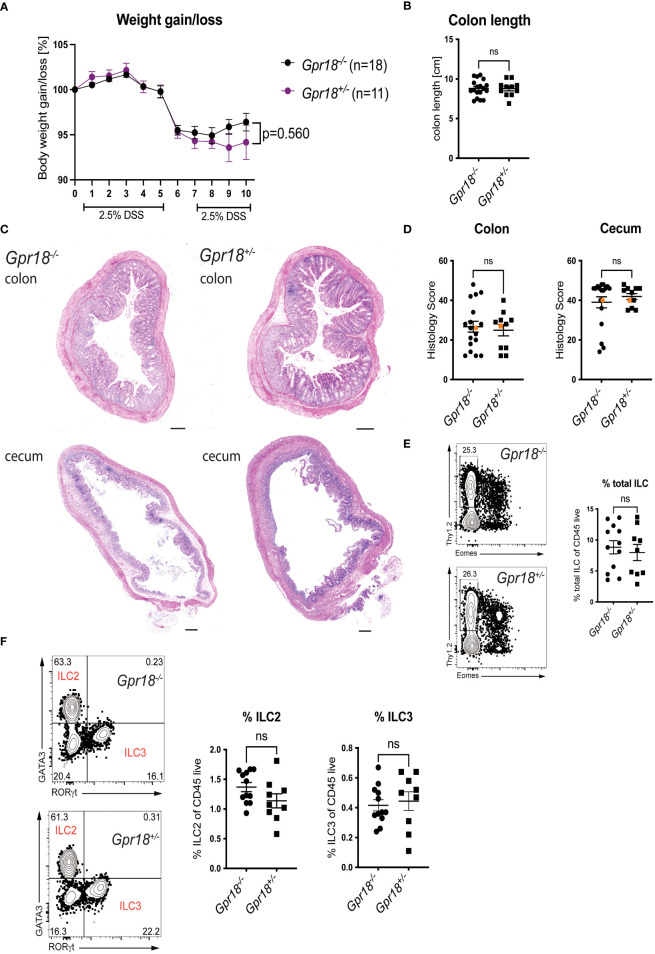
GPR18 does not play a role in the DSS model of colitis. Colitis was induced in *Gpr18^-/-^* and control littermates (*Gpr18^+/-^*) by two cycles of 2.5% DSS for 5 days followed by two days of water and disease progression was monitored. **(A)** Mice were weighed daily for 10 days and bodyweight change as % starting weight was determined. Data was combined from three independent experiments. **(B)** Colon lengths at end point. **(C)** H&E staining of representative distal colon and cecum sections from at 40x (scale bar 200 μm) magnification. **(D)** Blinded histology scores from distal colon and cecum combined from 3 independent experiments. The representative images were taken from mice represented by the orange colored data points. **(E)** Percentage of total ILC isolated from the colon lamina propria from two independent DSS colitis experiments. Total ILC were gated as Thy1.2^+^CD45^+^LIN^-^CD3^-^, Lineage markers were CD19, B220, CD11c and Gr1. **(F)** Percentage of ILC2 and IlC3 isolated from the colon lamina propria from two independent DSS experiments. ILC2 were gated as CD45^+^LIN^-^CD3^-^Thy1.2^+^Eomes^-^GATA3^+^ and ILC3 were gated as CD45^+^ LIN^-^CD3^-^Thy1.2^+^Eomes^-^RORγt^+^. Error bars represent S.E.M. Two-way ANOVA (Tukey’s test) **(A)** and nonparametric T-test (Mann-Whitney test) **(B, D–F)**. ns, not significant.

*Gpr18* expression was increased in the colon in the DSS model of colitis, a model that depends on innate immune responses (**Figure S4A**). Therefore, we compared *Gpr18^-/-^* vs. control littermates (*Gpr18^+/-^*) after subjecting mice to two cycles of DSS of 5 days each, with two days of water in between, as previously described (26). Disease progression was monitored by body weight loss. While there was a trend suggesting the absence of GPR18 might be protective, this did not reach statistical significance (**Figure 3A**). The colon length, a sign of inflammation and fibrosis, also was not significantly different between *Gpr18^-/-^* and control littermates (**Figure 3B**). Likewise, the histology scores comparing *Gpr18^-/-^* and control mice were similar in both the colon and cecum (**Figures 3C, D**). *Gpr18* is expressed by ILC2 and ILC3 in the small intestine (Immgen database) in steady-state and therefore we compared the percentages of total colonic ILC and ILC subsets in DSS-treated mice. Cells from the colon lamina propria were isolated as previously described (27). Percentages of total ILC and ILC subsets were similar in both genotypes (**Figures 3E, F**, **S4B**). In summary, the data indicate that GPR18 does not play a nonredundant role in the DSS-induced model of colitis.

### GPR18 expression by T cells is not required for colitis

**Figure 4:**
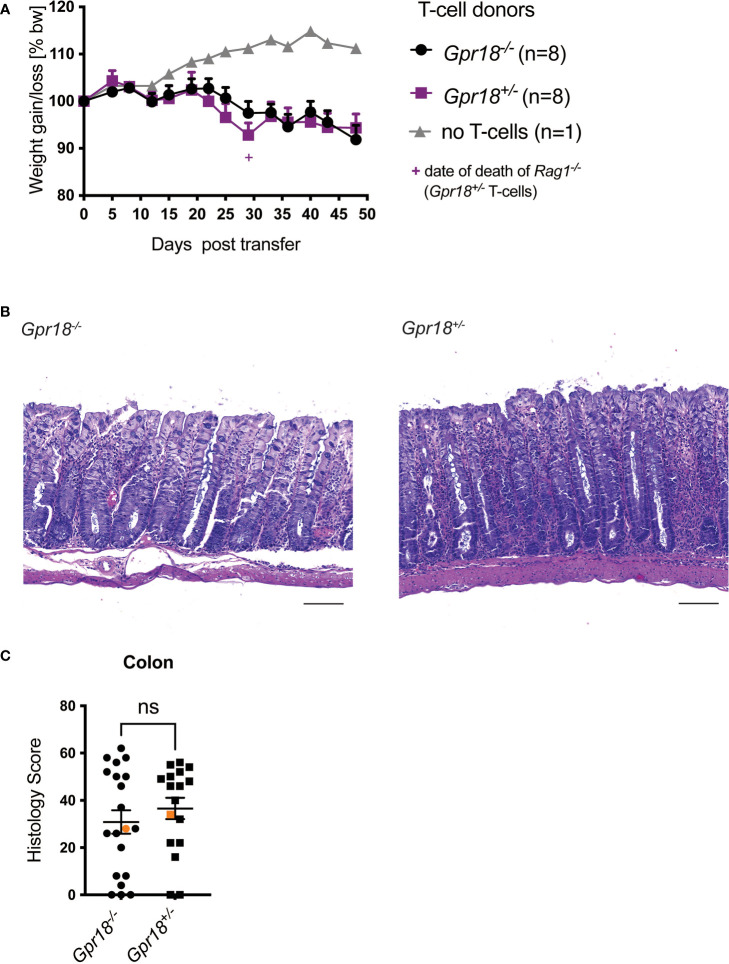
GPR18 expression by T cells is not required for colitis in the transfer model. Colitis was induced by adoptive transfer of GPR18 deficient (*Gpr18^-/-^*) vs. littermate control (*Gpr18^+/-^*) naive CD4^+^ T cells into *Rag1^-/-^* recipients. **(A)** Weight change was monitored as % starting body weight on the indicated days. A representative weight graph from one of 3 independent experiments is shown. The weight of dead mice was carried forward, indicated with a + sign in magenta for one mouse for *Gpr18^+/-^* T cells. **(B)** H&E staining of representative colon sections (middle part of whole colon) at 40x (scale bar 100 μm) magnification. **(C)** Blinded histology scores from the middle part of the whole colon of 3 independent experiments combined. Representative images were taken from mice represented by the orange colored data points. Error bars represent S.E.M. Two-way ANOVA (Tukey’s test) **(A)** and nonparametric T-test (Mann-Whitney test) **(C)**. ns, not significant.

We investigated the role of GPR18 in a second mouse colitis model, in this case initiated by adaptive immunity, because the effect could be model-dependent. We found the expression of *Gpr18* mRNA also was increased in the colon of mice in the T cell transfer model of colitis (**Figure S4B**), to an even greater extent than *Gpr183*. Moreover, GPR18 expression affects intraepithelial lymphocytes (IEL) subpopulations (15) and based on RNAseq data, *Gpr18* mRNA is expressed by mouse T cells (ImmGen database). Therefore, we transferred naïve T cells from *Gpr18^-/-^* vs. control littermates (*Gpr18^+/-^*) into *Rag1^-/-^* recipients and monitored disease progression. There was no significant difference in body weight loss (**Figure 4A**). Histopathologic analysis confirmed that *Gpr18^-/-^* and control littermates were not different in terms of disease parameters (**Figures 4B, 4C**). Taken together, GPR18 expression by T lymphocytes is not required for the transfer model of colitis.

### GPR18 expression by innate cells is not required for T cell transfer colitis

GPR18 is expressed on innate immune cells, for example by ILC2 and ILC3 in the small intestine (ImmGen database). Therefore, we tested if GPR18 expression by cells other than T lymphocytes, including innate immune cells in the *Rag1^-/-^* hosts, was required for colitis.

We crossed *Gpr18^-/-^* and control littermates onto a *Rag1^-/-^* background and injected naïve CD4^+^ T cells from wildtype C57BL/6 donors into *Rag1^-/-^Gpr18^-/-^* and control littermates (*Rag1^-/-^Gpr18^+/-^*).

**Figure 5:**
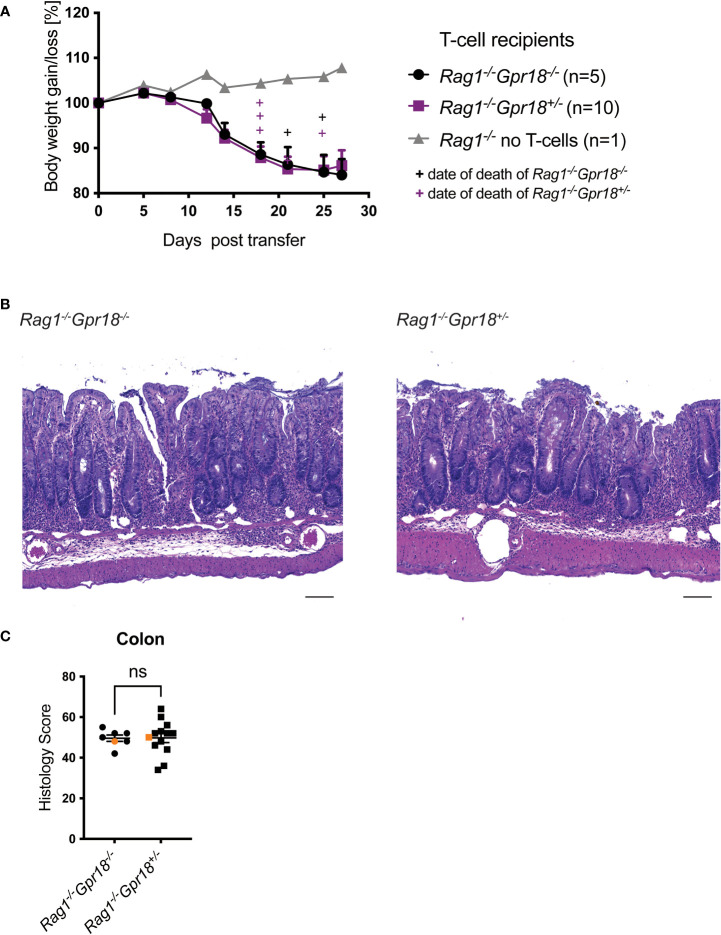
GPR18 does not play a role on innate cells in the T cell transfer host. Colitis was induced by adoptive transfer of naive CD4^+^ T cells from C57BL/6 mice into GPR18 deficient (*Gpr18^-/-^*) vs. control (*Gpr18^+/-^*) *Rag1^-/-^* recipients. Disease progression was monitored. **(A)** Weight change was monitored 2-4 times per week as % starting body weight. A representative weight graph of 3 independent experiments is shown. The weight of dead mice was carried forward, indicated with + signs in magenta for control and in black for *Rag1^-/-^Gpr18^+/-^* mice. **(B)** H&E staining of representative colon sections (middle part of whole colon) at 40x (scale bar 100 μm) magnification. **(C)** Blinded histology score from the middle part of whole colon of 3 independent experiments combined. Representative images were taken from mice represented by the orange colored data points. Error bars represent S.E.M. Two-way ANOVA (Tukey’s test) **(A)** and nonparametric T-test (Mann-Whitney test) **(C)**. ns, not significant.

We did not observe a significant difference between *Rag1^-/-^* mice based on the *Gpr18* genotype in terms of body weight loss (**Figure 5A**). Histopathology analysis also did not reveal a difference between *Rag1^-/-^Gpr18^-/-^* and *Rag1^-/-^Gpr18^+/-^* mice (**Figures 5B, C**). Therefore, although *Gpr18* expression was increased in the colon in colitis patients and in two mouse models of colitis, expression of GPR18, like as for GPR183, does not play a role in regulating disease severity.

## Discussion

Several hundred SNPs have been identified by GWAS as susceptibility loci for IBD, but it cannot be assumed that these act in *cis* to affect the expression of nearby genes. Therefore, we focused on the genes that were found to be related to these IBD GWAS hits, and asked which of them showed increased expression in colonic tissues of patients with UC and also in patients with Crohn’s disease, in order to identify processes that reflect both types of intestinal inflammation. A small list of genes emerged, including genes for chemokines and chemokine receptors, cytokine receptors, signaling molecules and transcription factors. We focused on *GPR183*, with SNP rs9557195 located in an intron of the gene and increased transcripts in colon tissue from both types of IBD patients. GPR183 had not been implicated in IBD studies at the time this work began, but it had been found to influence B lymphocyte localization during the germinal center response (25), and was shown to mediate migration of T cells into the inflamed CNS in an experimental autoimmune encephalomyelitis model (9). Furthermore, we found that the expression of *GPR183* mRNA was increased in patients resistant to TNF blockade therapies compared to responders, and *Gpr183* expression was increased in colon tissue in the T cell transfer model of colitis. Analyzing published scRNA-seq data, we found increased expression of *GPR183* transcripts in colonic lymphocytes from patients with UC, but not in their PMBCs. It is possible that *GPR183* transcripts also are increased in other colonic cell types. Our conclusions agree with a previously published study carried out while this study was ongoing. They found that *GPR183* mRNA as well as *CH25H* and *CYP7B1* were increased in colon biopsy samples of UC patients vs. healthy controls (10), although Crohn’s disease patients and responsiveness to TNF blockade were not included in that analysis. This group also detected increased *Gpr183*, *Ch25h* and *Cyp7b1* expression in the acute and chronic DSS colitis models, but they did not observe an effect of *Gpr183* or *Ch25h* gene deficiency on inflammation severity (10).

Based on the GWAS and gene expression studies, we analyzed the role of GPR183 in the T cell transfer model of colitis. We did not find an effect of GPR183 on disease severity when *Gpr183* was deleted in either the donor T cells or on other cell types in the *Rag1^-/-^* cell transfer hosts. We also studied the role of the most potent GPR183 ligand, by using *Rag1^-/-^* recipient mice lacking the relevant enzyme CH25H. There was no difference between *Rag1^-/-^Ch25h^-/-^* and littermate controls. It is possible that the synthesis of an alternative ligand, 7α,27-diHC, by the enzyme Cyp27a1 (32) becomes more prominent by a compensatory mechanism when CH25H is not expressed. However, it was reported that the expression of *Cyp27a1* was not increased in several colitis models when *Ch25h* was deleted (10, 33). Also, we have not excluded the possibility that deletion of *Gpr183* on both the donor T cells and the *Rag1^-/-^* hosts would have had an effect on colitis induction. Furthermore, although a comparison of heterozygotes to homozygous knockouts is the most rigorous one possible, it could miss smaller effects on colitis pathogenesis. Our results agree with those from a study showing *Gpr183^-/-^* mice were not different from wild type controls in a DSS model of colitis (10). Our results are in contrast, however, to other findings suggesting GPR183 is relevant in some colitis models in which disease is initiated by stimulating the innate rather than the adaptive immune system. In colitis induced by exposure to a low dose of DSS, *Il10^-/-^Gpr183^-/-^* animals had less severe inflammation compared to *Il10^-/-^* controls (10). Furthermore, in the model induced by injection of an anti-CD40-mAb in *Rag1^-/-^* mice, inflammatory foci were reduced in *Rag1^-/-^Gpr183^-/-^* mice due to decreased mobilization of group 3 innate lymphoid cells (ILC3s) from colonic patches into epithelial-adjacent tissue (5). These findings indicate a moderately pro-inflammatory role of GPR183 expression, perhaps acting in ILC, in some models when the innate immune system is hyper-stimulated. The results implicating GPR183 or not will vary due to differences in the microbiome and environments in different colonies, but also due to the different methods by which intestinal inflammation is initiated.

A limitation of GWAS studies is that they usually do not provide a direct link to a specific gene or cell type. Therefore, we also analyzed the effect of lack of expression of *GPR18*, which is in relatively close proximity to SNP rs9557195 and which is relevant for some aspects of mucosal immunity. GPR18 was shown to be involved in regulating the number and activation state of TCRγδ lymphocytes in the small intestine epithelium (15). The distribution of CD8α^+^ T lymphocytes might be relevant for IBD pathogenesis (34). In a mouse model of polymicrobial sepsis, GPR18 was involved in the resolution phase of inflammation, with the pro-resolving effects augmented and survival increased with treatment with a putative GPR18 ligand, resolvin D2 (23). Furthermore, the GPR18 small molecule agonist PSB-KK-1415 reduced visceral pain in a chronic colitis model induced by TNBS (35). A recent study showed that *Gpr18* mRNA expression was increased in IL23R competent Th1-like cells in mice, correlative data suggesting that GPR18 may be a critical mediator of pathogenicity by IL-23R in colitogenic Th1-like cells (36)

Like *GPR183*, *GPR18* transcripts increased in microarray expression data from colon biopsies of UC and Crohn’s disease patients versus healthy controls and in colon tissue from mouse models of colitis. scRNA-seq data showed *GPR18* transcripts were increased in the colonic B cells from IBD patients. We investigated the role of GPR18 in two mouse colitis models, the DSS and the T cell transfer model. We found that GPR18 expression is not essential for influencing disease severity of colitis in mice, when its expression was deleted in all cell types in the DSS model, or in the transfer model when either T cell expression or expression in other cell types was eliminated.

A recently completed clinical trial compared *GPR183* expression in PBMCs of IBD patients with the CC (minor, imparts IBD risk) and TT (major) alleles of the rs9557195, relevant SNP. Patients with the CC-allele had a higher *GPR183* expression in naïve B cells compared to patients with the TT-allele, but no difference was observed in CD4^+^ and CD8^+^ naïve T cells. Cell types in the peripheral blood may not be the relevant types when analyzing for a quantitative effect on gene expression, which also was reflected by our analysis of scRNA-seq data. Importantly, there was not a significant correlation between SNP rs9557195 genotype and disease severity among this group of IBD patients (37). Overall, the data here and other studies indicate that expression of *GPR183*, and to a lesser extent *GPR18*, are biomarkers for the extent of intestinal inflammation that may act in several cell types. However, our data and the clinical data do not support a non-redundant role for the expression level of these GPRs in influencing the severity of colitis pathogenesis. The SNP rs9557195, however, could affect the expression of other genes in *cis* or *trans* relevant for colitis.

## Data availability statement

The datasets presented in this study can be found in online repositories. The names of the repository/repositories and accession number(s) can be found below: https://www.ncbi.nlm.nih.gov/geo/, GSE16879 https://www.ncbi.nlm.nih.gov/geo/, GSE22307 https://www.ncbi.nlm.nih.gov/geo/, GSE27302 https://www.ncbi.nlm.nih.gov/geo/, GSE125527.

## Ethics statement

The animal study was reviewed and approved by La Jolla Institute for Immunology Animal Care and Use Committee.

## Author contributions

MD designed and performed experiments and wrote the manuscript. D.G. designed experiments and performed data analysis. YL, DG, GV, VC, GA-G, T-FC, TP-J provided technical expertise, performed experiments and revised the manuscript. HC provided supervision and revised the manuscript. MK supervised the study and wrote the manuscript. All authors contributed to the article and approved the submitted version.

## Funding

NIH grant P01 DK46763 (MK), FWF Schroedinger Fellowship J4308-B34 (MD), NIH F32 AI 140581 (DG), a grant from the Praespero foundation (HC), Academia Sinica-UC San Diego Talent Development Program (T-FC).

## Acknowledgments

We thank D. Hinz and C. Dillingham for assisting with naïve CD4 T cell sorting; A. Denn and K. Dobaczewska for assisting with preparation of histology slides; J. Cyster for providing *Gpr183^-/-^* and *Gpr18^-/-^* mice; K. Kim and P. Ernst for histopathology expertise; K.Suchey, M.Hockaday and A. Landgrave for technical assistance.

## Conflict of interest

The authors declare that the research was conducted in the absence of any commercial or financial relationships that could be construed as a potential conflict of interest.

## Publisher’s note

All claims expressed in this article are solely those of the authors and do not necessarily represent those of their affiliated organizations, or those of the publisher, the editors and the reviewers. Any product that may be evaluated in this article, or claim that may be made by its manufacturer, is not guaranteed or endorsed by the publisher.
