# Supplementary material for: CD4+-mediated colitis in mice is independent of the GPR183 and GPR18 pathways
Source: Front Immunol. 2022 Oct 28;13:1034648. doi: 10.3389/fimmu.2022.1034648 (PMC9652117; doi:10.3389/fimmu.2022.1034648)
Supplement: Supplementary file 1 [file DataSheet_1.docx]

***Supplementary material***

**Supplemental Figure 1:**

***GPR183* expression in IBD patients and mouse colitis models.** A) A select list of genes based on GWAS hits that are significantly upregulated in Crohn’s disease patients (GEO data set: GSE16879). Data from human whole genome microarray, colon tissue of controls (n=6) and inflamed colon tissue in Crohn’s disease patients (n=17), all non-treated, was analyzed and log_2_ fold change was calculated. B) A select list of genes as above that are significantly upregulated in UC patients (GEO data set: GSE16879). Data from human whole genome microarray, colon tissue of controls (n=6) and inflamed colon tissue in UC patients (n=24), all non-treated, was analyzed and log_2_ fold change was calculated. C) Data from a human whole genome microarray of 43 IBD patients (GEO data set: GSE16879) was analyzed regarding mRNA expression of *GPR183* in non-inflamed colon tissue of healthy volunteers (n =6 ) vs. inflamed colon tissue of UC (n=24) and Crohn’s disease (n=19) patients. Arbitrary units. Each symbol represents a donor. D) *GPR183* expression in responders to Infliximab treatment vs. non-responders in data set GSE16879. Arbitrary units. Each symbol represents a donor. E) Microarray data (GEO data set: GSE27302) from *Rag^-/-^* mouse colon tissue in response to T cell transfer at weeks 0, 2, 4 and 6 was analyzed for *Gpr183* expression, counts per million (CPM). Each symbol represents a mouse. Error bars represent S.E.M. ANOVA (Kruskal-Wallis test) (C and D), One-way ANOVA (E).

**Supplemental Figure 2:**

***GPR183* expression by single cells from patients and healthy controls.** A) Analysis of *GPR183* expression by cell types from PBMCs from UC patients versus healthy controls from scRNA-seq data (GSE125527). T cells include both CD4 and CD8 T lymphocytes. Normalized log-transformed counts. Each dot represents a cell. B) Analysis of *GPR183* expression in colon tissue (distal part) from UC patients versus healthy controls from scRNA-seq data. Normalized log-transformed counts. Each dot represents a cell.

**

**Supplemental Figure 3:**

***GPR18* expression in patients and healthy controls.**

A) Gene map of the human *GPR183* and *GPR18* gene loci on chromosome 13. SNP rs9557195 in an intron of *GPR183* is shown as a vertical blue line. *GPR183* RNA is shown as well as the alternative transcripts of *GPR18*. Figure was adapted from UCSC genome browser. B) Data from a human whole genome microarray of 43 IBD patients (GEO data set: GSE16879) was analyzed regarding mRNA expression levels of *GPR18* in non-inflamed colon tissue of healthy volunteers (n =6) vs. inflamed colon tissue of UC (n=24) and Crohn’s disease (n=19) patients. Arbitrary units. Each symbol represents a donor. C) *GPR18* expression in responders to Infliximab treatment vs. non-responders in data set GSE16879. Arbitrary units. Each symbol represents a donor. D) Analysis of *GPR18* expression by cell types from PBMCs from UC patients versus healthy controls from available scRNA-seq data (GSE125527). T cells include both CD4 and CD8 T lymphocytes. Normalized log-transformed counts. Each symbol represents a cell. E) Analysis of *GPR18* expression in colon tissue (distal part) from ulcerative colitis patients versus healthy controls from scRNA-seq data. Normalized log- transformed counts. Each symbol represents a cell. ANOVA (Kruskal-Wallis test) (B and C).


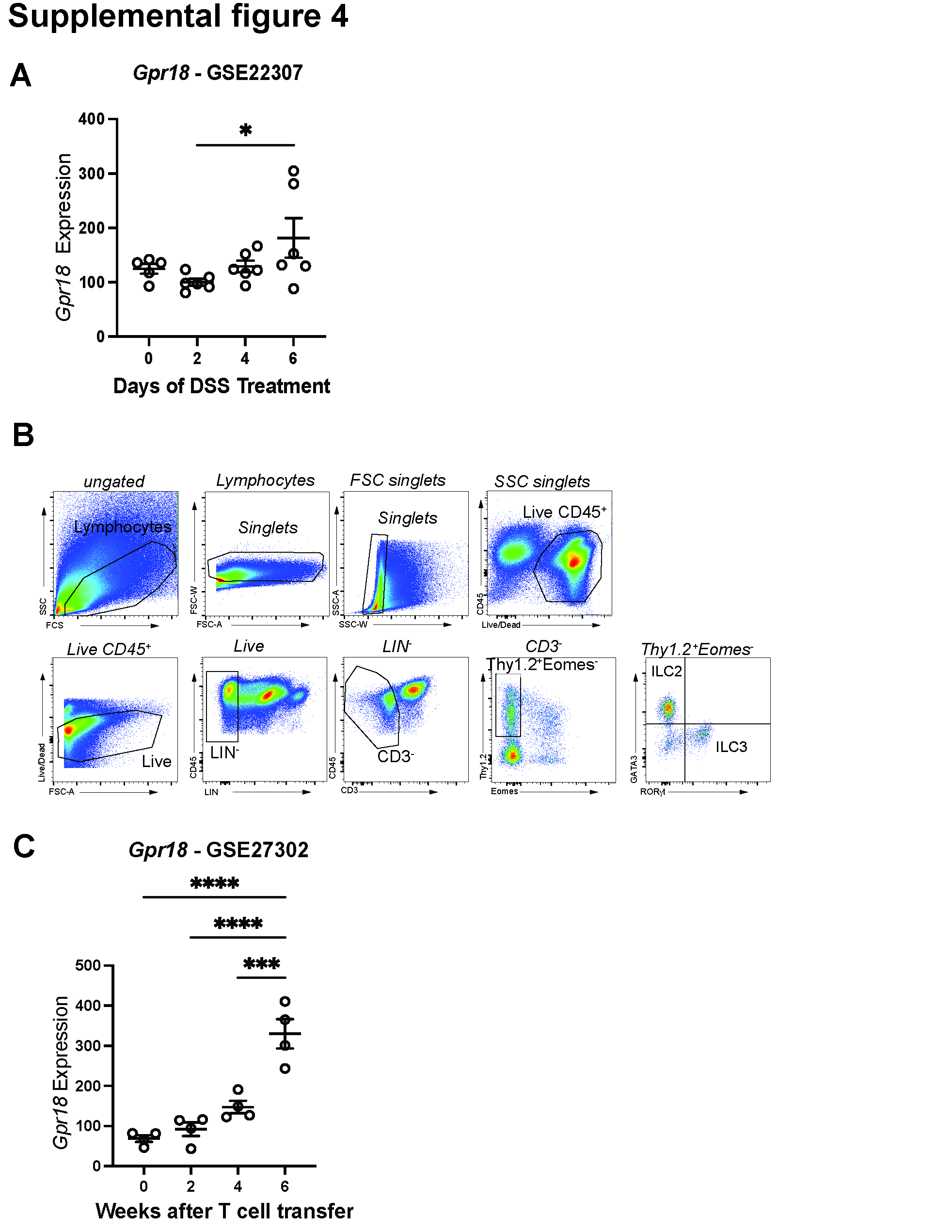


**Supplemental Figure 4**

***Gpr18* expression in mouse colitis models.**

A) Microarray data (GEO data set: GS22307) from mouse colon tissue in response to DSS at days 0, 2, 4 and 6 was analyzed regarding G*pr18* expression. CPM. Each symbol represents a mouse. B) ILC gating strategy. Lineage markers were CD19, B220, CD11c and Gr1. C) Microarray data (GEO data set: GSE27302) from *Rag^-/-^* mouse colon tissue in response to T cell transfer at weeks 0, 2, 4 and 6 was analyzed regarding *Gpr18* expression. CPM. Each symbol represents a mouse. One-way ANOVA (A and C).
